# Growth factor supportive care for chemotherapy-induced neutropenia suppresses antitumour immunity in checkpoint blockade-responsive pancreatic cancer

**DOI:** 10.1093/immadv/ltag013

**Published:** 2026-07-15

**Authors:** Brendan D Parent, Anthony E Kelly, Megan T Hoffman, Michael Dougan, Andrew J Aguirre, Stephanie K Dougan

**Affiliations:** Department of Medical Oncology, Dana-Farber Cancer Institute, Boston, MA, USA; Amherst College, Amherst, MA, USA; Department of Cancer Immunology and Virology, Dana-Farber Cancer Institute, Boston, MA, USA; Department of Immunology, Harvard Medical School, 350 Longwood Ave, Boston, MA 02115, USA; Department of Medicine, Division of Gastroenterology, Massachusetts General Hospital, Boston, MA, USA; Department of Medicine, Harvard Medical School, Boston, MA, USA; Department of Medical Oncology, Dana-Farber Cancer Institute, Boston, MA, USA; Department of Medicine, Harvard Medical School, Boston, MA, USA; Department of Cancer Immunology and Virology, Dana-Farber Cancer Institute, Boston, MA, USA; Department of Immunology, Harvard Medical School, 350 Longwood Ave, Boston, MA 02115, USA

**Keywords:** pancreatic cancer, G-CSF, neutrophils, checkpoint blockade, filgrastim, pegfilgrastim

## Abstract

**Introduction:**

Growth factors, including granulocyte colony-stimulating factor (G-CSF; pegfilgrastim, filgrastim), are used for prophylaxis or treatment of chemotherapy-induced neutropenia, yet their effects on antitumour immunity remain incompletely understood. We previously found that serum from patients with pancreatic ductal adenocarcinoma (PDAC) treated with multiagent chemotherapy plus G-CSF drove differentiation of T cell-suppressive monocytes *in vitro*, suggesting that supportive care interventions may shape immune responses in this disease.

**Methods:**

We evaluated the immunologic and therapeutic impact of G-CSF in two murine PDAC models that differ in their baseline frequencies of infiltrating T cells and in their responsiveness to checkpoint blockade immunotherapy.

**Results:**

In poorly immunogenic, T-cell-low tumours, use of G-CSF did not affect tumour growth or response to chemo- or immunotherapy, although neutrophil recovery was improved in mice receiving FOLFIRINOX and G-CSF compared to chemotherapy alone. In immunogenic tumours with a robust endogenous T-cell response, combination anti-PD1 and anti-CTLA-4 therapy resulted in durable tumour clearance. Combination with G-CSF diminished the effectiveness of checkpoint blockade and resulted in significantly fewer cured mice.

**Conclusion:**

G-CSF, commonly used for supportive care with FOLFIRINOX and other chemotherapy regimens, induces systemic immune suppression that can reduce the efficacy of T-cell-targeting immunotherapies.

## Graphical Abstract

**Graphical Abstract:**
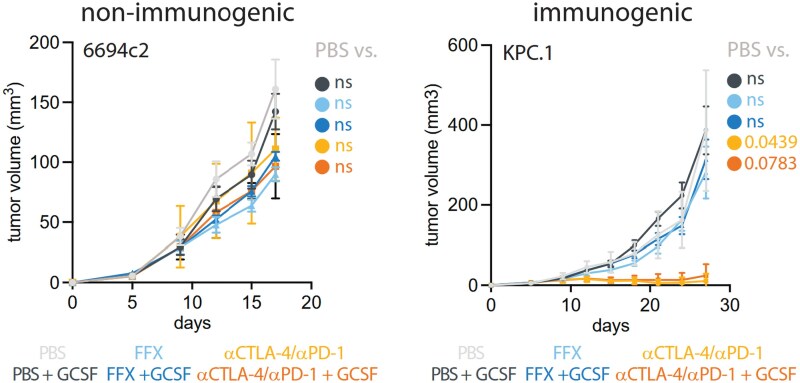

## Introduction

Pancreatic ductal adenocarcinoma (PDAC) is among the most lethal malignancies. The five-year survival rate of the disease is approximately 13%, with roughly half of patients presenting with metastasis at the time of diagnosis [1]. Multiagent chemotherapy regimens are the mainstay of first-line treatment and include FOLFIRINOX (a combination of folinic acid [leucovorin], fluorouracil [5-FU], irinotecan, and oxaliplatin), NALIRIFOX [2] (liposomal irinotecan, oxaliplatin, leucovorin, and fluorouracil), or gemcitabine/nab-paclitaxel [3]. FOLFIRINOX or NALIRIFOX are generally preferred first-line options for patients able to tolerate the more intensive therapy [4, 5]. Combining additional agents with intensive regimens such as FOLFIRINOX is inherently challenging because of its substantial toxicity. Grade 3 or 4 neutropenia is common, with a smaller subset experiencing febrile neutropenia [3]. Prophylactic or therapeutic administration of pegfilgrastim, a long-acting formulation of granulocyte colony–stimulating factor (G-CSF), is used to increase neutrophil production in the bone marrow, allowing for continued use of multiagent chemotherapy [6]. While essential for maintaining neutrophil counts and enabling chemotherapy delivery, G-CSF induces immunosuppressive neutrophils with myeloid-derived suppressor cell (MDSC)-like properties in both humans and mice [7]. Our group previously demonstrated that neutrophils generated in response to G-CSF can directly suppress T-cell proliferation *in vitro* and induce systemic immune suppression [7]. In combination with immunotherapy, such immune suppression could limit the therapeutic potential of immune checkpoint inhibitors by dampening antitumour T-cell responses.

Immune checkpoint inhibitors (ICIs) have transformed the treatment landscape for multiple cancer types. However, efforts to expand PDAC treatment to include ICIs have been largely unsuccessful, in part due to the immunosuppressive tumour microenvironment and low baseline T-cell infiltration characteristic of most pancreatic cancers [8–11]. Despite this, a small subset of patients shows benefit from immunotherapy, including those with high tumour mutational burden (TMB) arising from mismatch repair deficiency or other mutational processes, for which ICIs are FDA-approved regardless of histology [12, 13]. Although these mismatch repair–deficient (dMMR) tumours only account for approximately 1% of PDAC [14], a modestly larger fraction exhibits genomic instability driven by defects in DNA damage repair pathways [15–17]. PDAC harbouring mutations in homologous recombination repair genes has shown enhanced sensitivity to platinum-based chemotherapy and has supported the integration of PARP inhibitors into maintenance treatment strategies [18]. More recent clinical efforts have begun exploring whether deficiency in DNA damage repair genes PALB2, BRCA1, and BRCA2 can be leveraged to enhance antitumour immunity, including through combination approaches that incorporate immune checkpoint blockade [19, 20].

For patients with lower tumour mutational burden, therapeutic vaccination against personalized neoantigens [21, 22] or mutant KRAS [23, 24] may generate a robust pool of antitumour T cells capable of tumour rejection. Although vaccines are currently being trialled in disease settings where combination with multiagent chemotherapy can be avoided, this necessarily limits their utility. Finally, KRAS inhibition with daraxonrasib or other agents are showing impressive clinical responses and may have immune-potentiating side effects from limiting KRAS-driven production of immunosuppressive cytokines [25–27]. As combination immunotherapy strategies are increasingly explored in the setting of PDAC, the immunologic consequences of supportive care agents, including granulocyte colony-stimulating factor (G-CSF), warrant careful evaluation.

Here, we used two KPC-derived murine pancreatic cancer cell line models [28] with differing baseline immunogenicity to examine the impact of G-CSF-induced immune suppression in the context of chemotherapy and immune checkpoint blockade. These models include the poorly immunogenic, T-cell-low 6694c2 line [29–31], which is refractory to ICI therapy, and the immunogenic KPC.1 line [32, 33], which exhibits robust responsiveness to combined PD-1 and CTLA-4 inhibition. Using these systems, we characterized how G-CSF shapes systemic and tumour-associated myeloid populations and influences antitumour immune responses in settings where immune checkpoint blockade is effective. Human G-CSF cross-reacts with the mouse G-CSF receptor [34], allowing clinical-grade filgrastim to be used directly in mouse models. This approach allowed us to assess whether G-CSF alters therapeutic outcomes in pancreatic tumours capable of mounting endogenous T cell-mediated immunity.

## Materials and methods

### Animal models

All animal protocols were approved by Dana-Farber Cancer Institute’s Institutional Animal Care and Use Committee (IACUC) (protocol #14-019 and 14-037) and are in compliance with the NIH/NCI ethical guidelines for tumour-bearing animals. C57BL/6 mice were purchased from Jackson Labs (stock #000664).

### Cell lines

6694c2 cells were derived from a LSL-KrasG12D;p53+/floxed, Pdx-cre, YFP-floxed mouse and were gifted by Ben Stanger (University of Pennsylvania) [26]. KPC.1 were derived from a LSL-KrasG12D;p53+/floxed, Pdx-cre mouse and were gifted by Anirban Maitra (NYU). Cells were cultured at 37°C in RPMI media (Life Technologies) supplemented with 10% (v/v) inactivated foetal bovine serum, 2 mmol/l L-glutamine (Gibco), 1% (v/v) penicillin/streptomycin (Gibco), 1% (v/v) MEM nonessential amino acids (Gibco), 1 mmol/l sodium pyruvate (Gibco), and 0.1 mmol/l β-mercaptoethanol (Sigma).

### Subcutaneous tumour models

A total of 250 000 6694c2 suspended in HBSS or 400 000 KPC.1 suspended in HBSS with 10% Matrigel (Corning) were inoculated subcutaneously into the flanks of C57BL/6J mice. Mice were then randomized to receive PBS, PBS and G-CSF, FOLFIRINOX, FOLFIRINOX and G-CSF, aCTLA-4 and aPD-1, or aCTLA-4 and aPD-1 and G-CSF. FOLFIRINOX (5 mg/kg oxaliplatin, 50 mg/kg irinotecan, 75 mg/kg leucovorin, and 75 mg/kg 5-FU) was administered intravenously as previously described [35]. Antibodies (150 μg/mouse) and G-CSF (20 µg/mice, Neupogen) were administered by intraperitoneal injection. Tumour growth was monitored by calliper every 3 days until tumour harvest (for flow cytometry) or until mice reached endpoint due either to tumour volume or ulceration (for survival analysis).

### Agents

Mice were dosed with the following agents. Phosphate buffered saline (PBS, Gibco cat#10010-023); anti-CTLA-4 (clone 9D9, BioXcell); anti-PD-1 (clone RMP1-14, BioXcell); isotype control (clone LTF-2, BioXcell). Filgrastim (Neupogen); Fluorouracil injection (Fresenius Kabi); Oxaliplatin injection (Sandoz); Leucovorin Calcium injection (Fresenius Kabi); Irinotecan Hydrochloride injection (Hikma); Gemcitabine Injection (NovaPlus); n(ab)paclitaxel (Abraxane, Celgene) were obtained from the Dana-Farber Cancer Institute Pharmacy.

### Flow cytometry

Bone marrow was flushed from the femurs of treated mice using sterile PBS and strained through a 40-micron cell strainer. Spleens were crushed through a 40-micron cell strainer and lysed with ACK lysis buffer prior to resuspension in PBS. Tumours were harvested from treated mice at Days 14 or 20. Tumours were macerated and digested in collagenase IV (Sigma #C5138) and soybean trypsin inhibitor (Life Technologies #17075029). Tumour chunks were removed via filtration with a 40 μM filter, and the resulting single-cell suspension was washed with PBS. The single-cell suspensions from each tissue site were washed in ice-cold FACS buffer and then stained in FACS buffer (PBS with 2% heat-inactivated foetal calf serum and 2 mM EDTA). Stains were composed of B220 BV605 (BioLegend #103243), CD4 BV510 (BioLegend #100553), CD8α Pacific Blue (BioLegend #100725), CD11b FITC (BioLegend #101206), CD11c APC (BioLegend #117310), CD45 BV711 (BioLegend #103147), CD103 PE (BioLegend #121405), CD170 (Siglec-F) BV421 (BioLegend #155509), Gr-1 PE-Cy7 (BioLegend #108416), and Ly-6C BV570 (BioLegend #128030). Samples were analysed by spectral flow cytometry using a Sony SP6800 flow cytometer. Data were analysed using FlowJo (v10.8.1_CL)

### Statistical analysis

Pairwise, group comparisons and correlation analyses were performed using the Wilcoxon matched-pairs signed rank test, Brown–Forsythe and Welch ANOVA tests with Dunnett’s T3 multiple comparisons, Kruskal–Wallis test, Mann–Whitney test, log-rank (Mantel–Cox) test, chi-square test, and Pearson coefficient calculation. GraphPad PRISM version 10 was used for statistical analysis.

### Data availability

All data generated in this study are presented in the figures and supplementary data. Additional information may be provided upon request.

## Results

**Figure 1:**
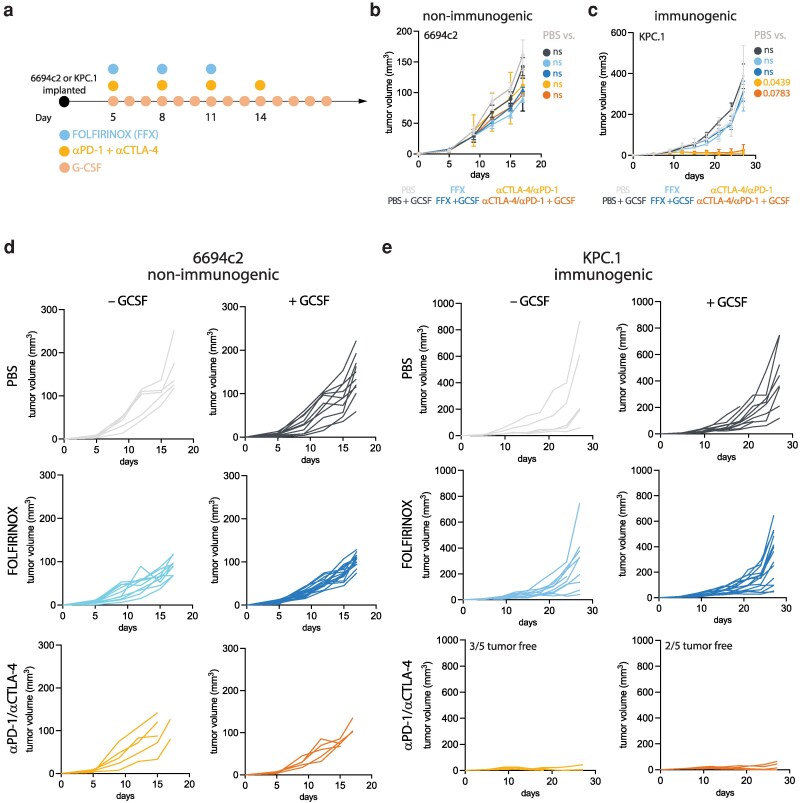
G-CSF reduces the efficacy of checkpoint blockade in immunogenic PDAC but has minimal impact in poorly immunogenic tumours. (a) Experimental design and treatment schema. C57BL/6 mice were subcutaneously implanted with 250 000 poorly immunogenic 6694c2 cells or 400 000 immunogenic KPC.1 pancreatic ductal adenocarcinoma cells. On Day 5 postimplantation, mice were randomized and received PBS, FOLFIRINOX (FFX; Days 5, 8, and 11), combined anti–PD-1 plus anti–CTLA-4 (αCTLA-4/αPD-1; 150 μg/mouse each antibody on Days 5, 8, 11, and 14), and/or granulocyte colony–stimulating factor (G-CSF, 20 μg/mouse). (b and c) Tumour growth curves of mice bearing 6694c2 (b) or KPC.1 (c) tumours treated with PBS (*n* = 5), PBS + G-CSF (*n* = 10), FFX (*n* = 10), FFX + G-CSF (*n* = 15), αCTLA-4/αPD-1 (*n* = 5), or αCTLA-4/αPD-1 + G-CSF (*n* = 5). Mean tumour volume +/− SEM. Statistical comparisons were performed using a one-way ANOVA with Kruskal–Wallis test on AUC values versus PBS controls. ns, not significant. (d and e) Individual tumour growth trajectories for mice shown in B and C. The fraction of tumour-free mice in the αCTLA-4/αPD-1 +/− G-CSF groups is indicated for KPC.1 tumours.

We evaluated responses to chemotherapy and immune checkpoint blockade in mice bearing 6694c2 or KPC.1 tumours (Fig. 1a). FOLFIRINOX was associated with modest effects on tumour growth in both models (Fig. 1b–c), with similar tumour progression observed in mice receiving chemotherapy alone or in combination with G-CSF. Mice inoculated with the poorly immunogenic, T-cell-low 6694c2 tumours were resistant to PD-1 and CTLA-4 blockade, with tumour growth similar to vehicle-treated controls regardless of whether G-CSF was administered (Fig. 1d). In contrast, the immunogenic, T-cell-high KPC.1 tumors exhibited sensitivity to immune checkpoint blockade, with significantly reduced tumour growth and durable tumour control in a subset of treated mice (Fig. 1e). Among mice bearing KPC.1 tumours, 3 of 5 mice treated with checkpoint blockade alone achieved complete tumour clearance, whereas 2 of 5 mice treated with checkpoint blockade in combination with G-CSF achieved complete tumour clearance.

These data demonstrate that immune checkpoint inhibition can induce durable tumour control in mice bearing immunogenic KPC.1 tumours. Given the KPC.1 tumour response to checkpoint blockade, we next examined how G-CSF administration alters systemic immune populations in this immunotherapy-responsive setting.

**Figure 2:**
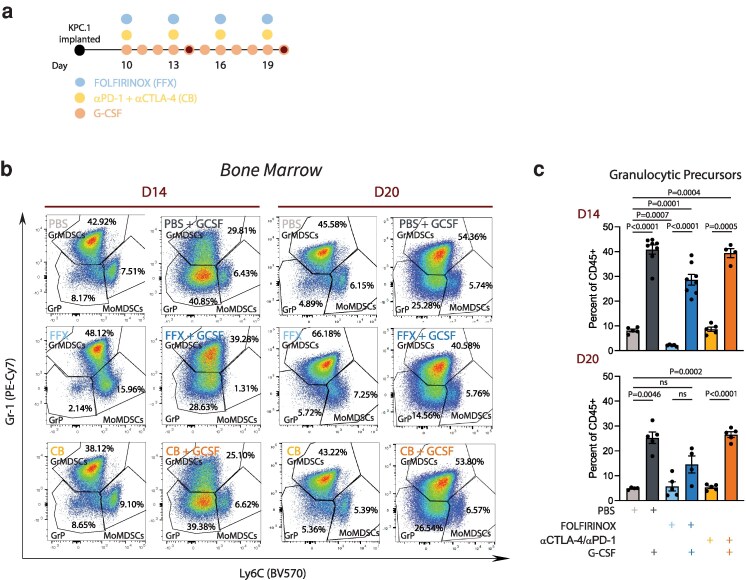
G-CSF promotes granulocytic progenitor expansion in bone marrow following chemotherapy and checkpoint blockade. (a) Experimental design. C57BL/6 mice were inoculated subcutaneously with 800 000 KPC.1 cells and treatment was initiated on Day 10 postimplantation with PBS, FOLFIRINOX (FFX; Days 10, 13, 16, and 19), or combined anti-PD-1 plus anti-CTLA-4 (αCTLA-4/αPD-1; 150 μg/mouse each antibody on Days 10, 13, 16, and 19), +/− daily G-CSF (20 μg/mouse) starting on Day 10. Bone m arrow was harvested on Days 14 (D14) or 20 (D20) for flow cytometric analysis. (b) Representative flow cytometry plots of bone marrow at D14 and D20 gated on CD45^+^ cells then displayed and gated by Gr-1 and Ly6C. GrP = granulocytic progenitors; GrMDSCs = granulocytic MDSCs; MoMDSCs = monocytic MDSCs. (c) Quantification of bone marrow granulocytic progenitors (GrP) at D14 and D20 as a percentage of CD45^+^ cells. Data represent mean +/− SEM. Dots show values from individual mice. Brown–Forsythe and Welch ANOVA followed by Dunnett’s T3 multiple comparisons test was used throughout. ns, not significant.

To assess the systemic effects of G-CSF, mice bearing established immunogenic KPC.1 tumours were treated with PBS, FOLFIRINOX, or combined anti–PD-1 and anti–CTLA-4 blockade, with or without G-CSF (Fig. 2a). Tumours were harvested at Days 14 or 20, corresponding to early and late treatment time points, and flow cytometry was used to assess systemic immune changes associated with G-CSF administration. The flow cytometric gating strategy used to define myeloid and lymphoid populations is shown in Supplementary Figure S1.

In the bone marrow, treatment with FOLFIRINOX resulted in a significant reduction in granulocytic precursor (GrP) cells at Day 14 (Figs. 2b–c). Across all treatment groups that received supplemental G-CSF, we observed a significant increase in granulocytic precursor cells relative to PBS-treated controls at Day 14 (Figs. 2b–c), and similar increases were maintained at Day 20. These results highlight that acute loss of proliferating GrP cells induced by FOLFIRINOX and rescue achieved with G-CSF can be recapitulated in mouse models.

**Figure 3:**
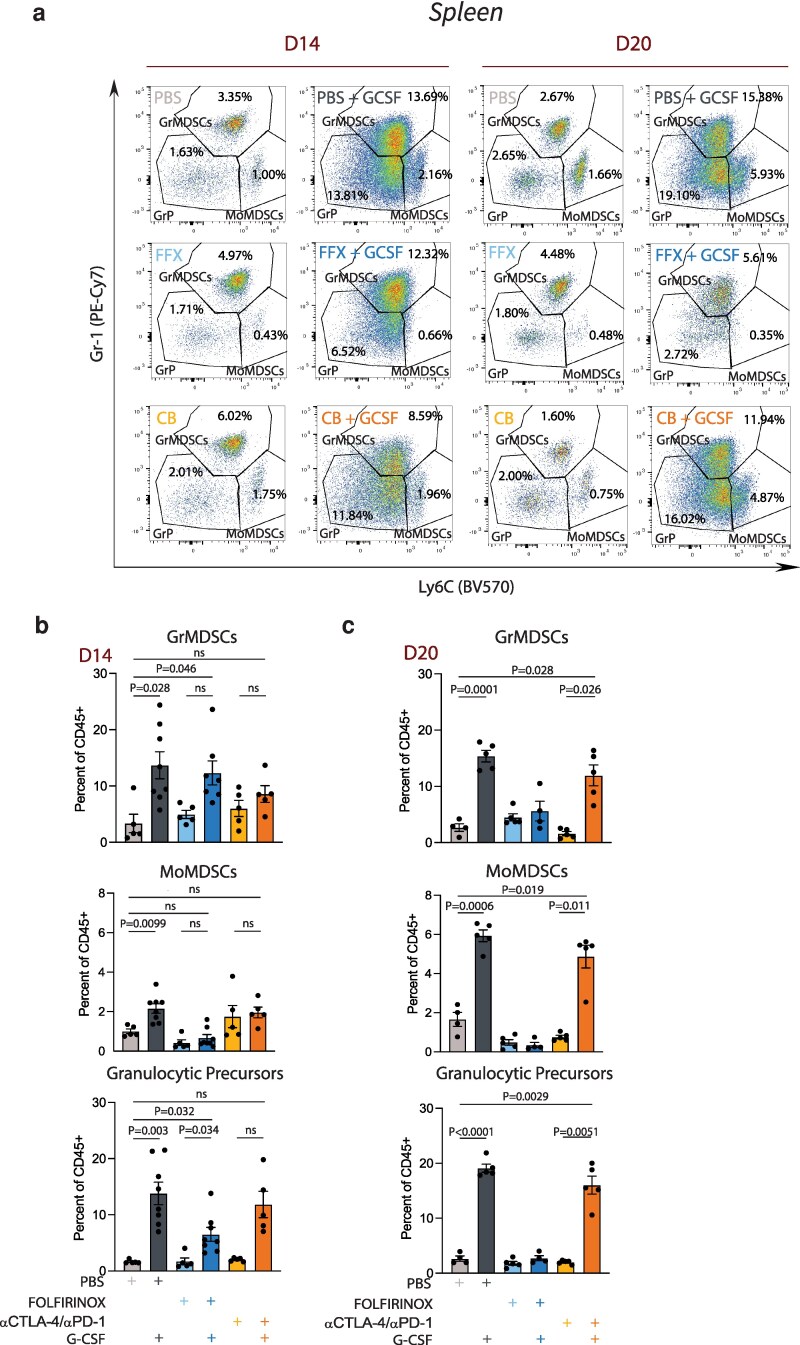
G-CSF promotes myeloid expansion in spleen following chemotherapy and checkpoint blockade. (a) Experimental conditions are as described in Fig. 2a. Spleens were harvested on Days 14 (D14) or 20 (D20) for flow cytometric analysis. Representative flow cytometry plots of spleen at D14 and D20 gated on CD45^+^ cells and then displayed and gated by Gr-1 and Ly6C. (b) Quantification of splenic GrMDSCs, MoMDSCs, and granulocytic progenitors at D14 as a percentage of CD45^+^ cells. (c) Quantification of splenic GrMDSCs, MoMDSCs, and granulocytic progenitors at D20 as a percentage of CD45^+^ cells. Data represent mean +/− SEM. Dots show values from individual mice. Brown–Forsythe and Welch ANOVA followed by Dunnett’s T3 multiple comparisons test was used throughout. ns, not significant.

In the spleen, a similar pattern of G-CSF–associated expansion of myeloid subsets was observed (Fig. 3a). In mice receiving immune checkpoint blockade plus G-CSF, GrMDSC levels were elevated at Day 14 and reached statistical significance at Day 20 (Fig. 3b–c). MoMDSCs were similarly increased at Day 20 in mice treated with checkpoint blockade and G-CSF, but not in mice receiving FOLFIRINOX and G-CSF. Expansion of granulocytic precursor populations followed trends similar to those observed for GrMDSCs. Collectively, these data indicate that G-CSF administration is associated with a systemic expansion of myeloid populations.

**Figure 4:**
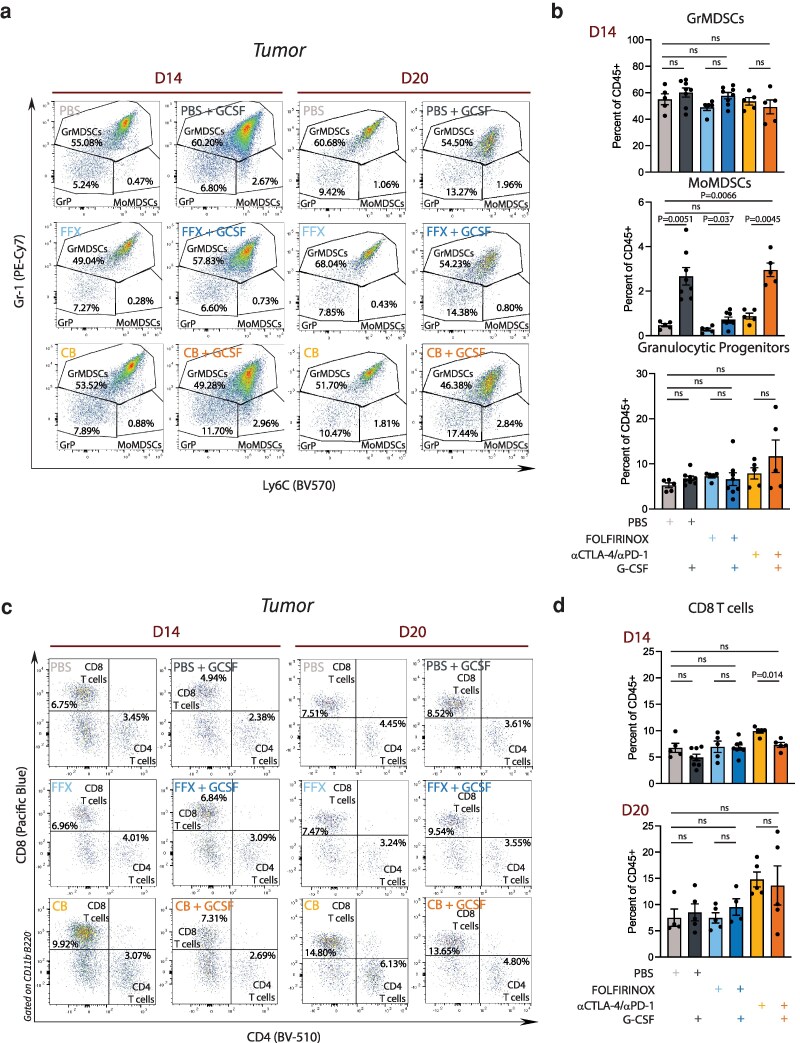
G-CSF remodels the tumour immune microenvironment by promoting MoMDSC accumulation and transiently reducing CD8^+^ T-cell infiltration. C57BL/6 mice were inoculated subcutaneously with 800 000 KPC.1 cells and treated beginning on Day 10 postimplantation with PBS, FOLFIRINOX (Days 10, 13, 16, and 19), or combined anti-PD-1 plus anti-CTLA-4 (αCTLA-4/αPD-1; 150 μg/mouse each antibody on Days 10, 13, 16, and 19), +/− daily G-CSF (20 μg/mouse). Tumours were harvested at D14 or D20 for flow cytometric analysis. (a) Representative flow cytometry plots of tumour-infiltrating myeloid populations at D14 and D20 gated on CD45^+^ cells and then displayed and gated by Gr-1 and Ly6C cells to identify GrP, GrMDSCs, and MoMDSCs. (b) Quantification of intratumoural GrMDSCs, MoMDSCs, and granulocytic progenitors expressed as a percentage of CD45^+^ cells on D14. (c) Representative flow cytometry plots of tumour-infiltrating T cells at D14 and D20 gated on CD45 ^+^ CD11b^−^B220^−^ cells and analysed by CD4 and CD8 expression to identify CD4^+^ and CD8^+^ T-cell subsets. (d) Quantification of intratumoural CD8^+^ T cells at D14 and D20 expressed as a percentage of CD45^+^ cells. Data represent mean +/− SEM. Dots show values from individual mice. Brown–Forsythe and Welch ANOVA followed by Dunnett’s T3 multiple comparisons test was used throughout. ns, not significant.

We then examined whether the systemic immune changes were reflected within the tumour immune compartment. Flow cytometric analysis of KPC.1 tumours revealed that granulocytic myeloid-derived suppressor cells (GrMDSCs) comprised the majority of CD45^+^ immune cells in the tumour microenvironment. Although GrMDSC frequencies were higher in tumours from mice treated with FOLFIRINOX plus G-CSF compared with FOLFIRINOX alone, this increase did not reach statistical significance (Fig. 4a–b). In contrast, monocytic myeloid-derived suppressor cell (MoMDSC) abundance was significantly increased in tumours from mice receiving G-CSF compared with those not receiving G-CSF (Fig. 4b). This increase was observed at both Days 14 and 20 across all treatment contexts, although the magnitude of the effect was more modest in the FOLFIRINOX-treated group.

Next, we assessed whether these changes in myeloid populations were associated with alterations in tumour-infiltrating T-cell abundance. Checkpoint blockade was associated with a modest increase in infiltrating CD8^+^ T cells, although this effect was mitigated by the addition of G-CSF, particularly at early time points (Fig. 4c–d). These findings suggest that G-CSF administration is associated with remodelling of the tumour microenvironment, including limited CD8^+^ T-cell accumulation and increased accumulation of monocyte-lineage cells.

**Figure 5:**
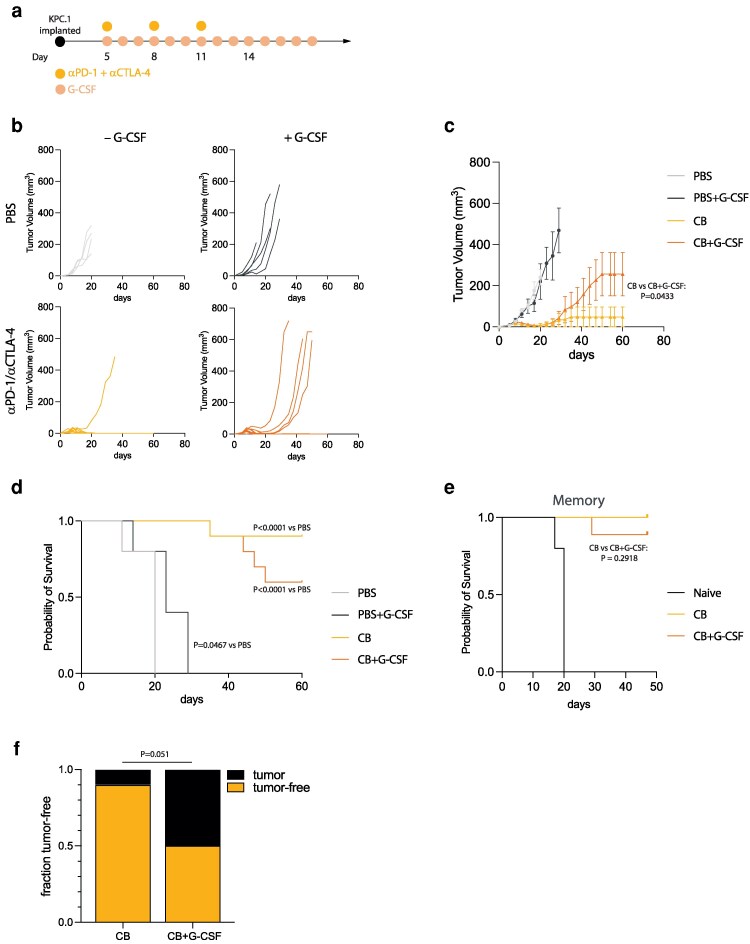
G-CSF impairs antitumour immunity in an immunogenic pancreatic cancer model. (a) Schematic of experimental design. C57BL/6 mice were inoculated subcutaneously with 800 000 KPC.1 cells and treated beginning on Day 5 postimplantation with PBS or combined anti-PD-1 plus anti-CTLA-4 (αCTLA-4/αPD-1; 150 μg/mouse each antibody on Days 5, 8, and 11), +/− daily G-CSF (20 μg/mouse). (b) Individual mouse tumour measurements tracked over time. (c) Combined tumour growth curves from mice shown in B, treated with PBS (*n* = 5), PBS + G-CSF (*n* = 5), αCTLA-4/αPD-1 (*n* = 10), or αCTLA-4/αPD-1 + G-CSF (*n* = 10). Mean tumour volume +/− SEM. Tumour growth curves were compared using a one-tailed Mann–Whitney test on tumour volume at the final time point. (d) Combined survival data from mice treated in A. Survival curves were compared using the log-rank (Mantel–Cox) test. Statistical comparisons are shown for each treatment group versus PBS. (e) Surviving mice from A were rechallenged on Day 60 with 800 000 KPC.1 cells implanted subcutaneously in the opposite flank and followed for survival. A control cohort of *n* = 5 C57BL/6 mice (naïve) was included. Statistical comparison of rechallenge outcome between groups was performed using the log-rank (Mantel–Cox) test. (f) Fraction of tumour-free versus tumour-bearing mice following primary and secondary challenge. Data from primary tumour clearance and rechallenge experiments were combined. Statistical comparison between αCTLA-4/αPD-1 and αCTLA-4/αPD-1 + G-CSF groups was performed using the chi-square test.

To assess whether G-CSF-associated immune remodelling corresponded with functional consequences for antitumour immunity, we evaluated tumour control and survival in mice bearing immunogenic KPC.1 tumours treated with combined PD-1 and CTLA-4 blockade in the presence or absence of G-CSF (Fig. 5a; *n* = 10 mice per group). Addition of G-CSF resulted in increased tumour burden compared to checkpoint blockade alone, with significantly greater tumour growth in the combination group (Fig. 5b–c). Both checkpoint blockade groups showed significantly improved survival compared to PBS-treated controls (Fig. 5d). Mice that were successfully cured of their tumours formed immunologic memory, as assessed by the prevention of tumour growth upon secondary challenge and in the absence of further therapy (Fig. 5e). There was no significant difference in memory between treatment with combined PD-1 and CTLA-4 blockade alone or in combination with G-CSF. When combining outcomes across primary and secondary tumour challenges, checkpoint blockade alone resulted in a higher proportion of tumour-free mice, with 9 of 10 mice achieving tumour clearance, whereas the addition of G-CSF reduced this fraction to 5 of 10 mice (Fig. 5f). These findings suggest that although G-CSF does not completely eliminate the tumour-clearing effects of immune checkpoint inhibition, it reduces the likelihood of achieving durable tumour clearance in this immunotherapy-sensitive setting.

## Discussion

Despite transformative advances in cancer immunotherapy, pancreatic ductal adenocarcinoma has shown limited clinical responsiveness to immune-based treatments. However, immune checkpoint inhibitors are increasingly being explored in biologically defined subsets of PDAC and in combination treatment strategies, heightening the importance of identifying factors that may influence antitumour immune responses in this disease. Here, we demonstrate that G-CSF, a supportive care agent routinely administered to enable delivery of intensive chemotherapy, can suppress antitumour immunity in PDAC models that mount an endogenous T-cell response. Using a subcutaneously implanted immunogenic KPC-derived tumour model responsive to immune checkpoint blockade, we show that G-CSF reduces the frequency of tumour clearance and modestly diminishes overall survival following combined PD-1 and CTLA-4 inhibition. In contrast, G-CSF has minimal impact in poorly immunogenic tumours that lack baseline responsiveness to immunotherapy. These results extend and functionally contextualize our prior observations that G-CSF induces systemic immune suppression in the setting of FOLFIRINOX chemotherapy. In earlier work, the immunologic consequences of G-CSF could not be linked to therapeutic outcomes due to the absence of an effective T-cell response in commonly used PDAC models [7]. Using an immunogenic PDAC model responsive to immune checkpoint blockade, we show that G-CSF-induced myeloid skewing directly impairs tumour control.

Mechanistically, our data suggest that G-CSF alters antitumour immunity primarily by reshaping the myeloid compartment rather than by directly affecting tumour-infiltrating T cells. Systemically, G-CSF administration expanded granulocytic precursors, consistent with its role in stress-induced granulopoiesis [36]. Within the tumour microenvironment, G-CSF preferentially increases monocytic MDSCs, while granulocytic populations remain largely unchanged. Notably, intratumoural CD8^+^ T-cell frequencies are largely preserved across treatment conditions, with only a modest early decrease observed following G-CSF administration. These findings align with established models of MDSC-mediated immune regulation, in which suppressive myeloid populations inhibit T-cell effector function without necessarily reducing T-cell abundance [37, 38].

The clinical relevance of these observations lies in the growing effort to identify biologically defined subsets of PDAC that may benefit from immunotherapy. While the majority of PDAC tumours are refractory to immune checkpoint blockade, tumours with increased antigenicity, including those harbouring defects in DNA damage repair pathways, may exhibit partial sensitivity to checkpoint inhibition [12, 19]. In these contexts, factors that modulate the immune landscape, including supportive care agents, become increasingly important determinants of therapeutic efficacy. Our data suggest that G-CSF, while essential for maintaining dose intensity and patient safety during intensive chemotherapy, may represent an underappreciated variable in immunotherapy combination strategies for PDAC.

Importantly, these findings do not suggest that G-CSF should be avoided in standard clinical practice. Rather, they highlight the need to account for the immunologic effects of supportive care interventions when designing and interpreting immunotherapy trials. Chemotherapy regimens that do not require routine G-CSF support, such as gemcitabine/n(ab)paclitaxel, alternative scheduling strategies, or careful patient selection based on immunogenic features, warrant consideration as immunotherapy is extended to PDAC and other traditionally resistant cancers.

This study has several limitations. The precise mechanisms through which G-CSF–expanded myeloid populations suppress antitumour immunity were not directly defined, and future work will be needed to clarify the relative contributions of these suppressive pathways. While prior literature supports the immunosuppressive activity of these populations, their functional capacity was not directly evaluated here. In addition, although two PDAC models with distinct immunogenic features were examined, these were subcutaneously implanted models, and further work across additional tumour systems, including in the orthotopic or liver metastatic setting, may help establish the broader generalizability of these findings. Finally, the impact of G-CSF in subsequent lines of therapy once immunologic memory has been established was not evaluated. Nonetheless, our results extend prior observations by establishing a functional link between G-CSF administration and impaired immunotherapy efficacy in PDAC.

In summary, our findings demonstrate that G-CSF is not immunologically neutral in the context of PDAC immunotherapy. As immune-based treatments are increasingly explored in PDAC, understanding how standard supportive care interventions interact with antitumour immunity will be essential for designing combination therapies and accurately interpreting clinical trial outcomes.

## Supplementary Material

ltag013_Supplementary_Data

## Data Availability

All data generated in this study are presented in the figures and supplementary data. Additional information may be provided upon request.
