# Supplementary material for: Growth factor supportive care for chemotherapy-induced neutropenia suppresses antitumour immunity in checkpoint blockade-responsive pancreatic cancer
Source: Immunother Adv. 2026 Jul 15;6(1):ltag013. doi: 10.1093/immadv/ltag013 (PMC13398997; doi:10.1093/immadv/ltag013)
Supplement: ltag013_Supplementary_Data [file ltag013_supplementary_data.zip › Supplementary_Legends.docx]

**Supplemental Figure 1. Flow cytometry gating scheme for bone marrow.**

(A) Gating scheme for bone marrow analysis of C57BL/6 mice from Figure 2. Bone marrow was harvested on day 14 (D14) or day 20 (D20) for flow cytometric analysis.

(B) Total CD11b^+^ cells were quantified. Each dot represents an individual mouse.

(C) Total eosinophils were quantified. Each dot represents an individual mouse.

(D) Total CD4^+^ cells were quantified. Each dot represents an individual mouse.

(E) Total CD8^+^ cells were quantified. Each dot represents an individual mouse.

(F) Total GrMDSCs were quantified. Each dot represents an individual mouse.

(G) Total MoMDSCs were quantified. Each dot represents an individual mouse.

(H) Total B cells were quantified. Each dot represents an individual mouse.

Data represent mean +/- SEM. Brown–Forsythe and Welch ANOVA followed by Dunnett’s T3 multiple comparisons test was used throughout. ns, not significant.

**Supplemental Figure 2. Flow cytometry gating scheme for spleen.**

(A) Gating scheme for spleen analysis of C57BL/6 mice from Figure 2. Spleen was harvested on day 14 (D14) or day 20 (D20) for flow cytometric analysis.

(B) Total CD11b^+^ cells were quantified. Each dot represents an individual mouse.

(C) Total CD4^+^ cells were quantified. Each dot represents an individual mouse.

(D) Total CD8^+^ cells were quantified. Each dot represents an individual mouse.

(E) Total B cells were quantified. Each dot represents an individual mouse.

Data represent mean +/- SEM. Brown–Forsythe and Welch ANOVA followed by Dunnett’s T3 multiple comparisons test was used throughout. ns, not significant.

**Supplemental Figure 3. Flow cytometry gating scheme for tumor infiltrates.**

(A) Gating scheme for tumor CD8 T cell quantification shown in Figure 3. Tumors were harvested on day 14 (D14) or day 20 (D20) for flow cytometric analysis.

(B) Total CD11b^+^ cells were quantified. Each dot represents an individual mouse.

(C) Total CD4^+^ T cells were quantified. Each dot represents an individual mouse.

(D) Total B cells were quantified. Each dot represents an individual mouse.

(E) Total GrMDSCs, MoMDSCs, and granulocytic precursor cells at day 20 (D20) were quantified. Each dot represents an individual mouse.

Data represent mean +/- SEM. Brown–Forsythe and Welch ANOVA followed by Dunnett’s T3 multiple comparisons test was used throughout. ns, not significant.
