# Supplementary figures and images for: Growth factor supportive care for chemotherapy-induced neutropenia suppresses antitumour immunity in checkpoint blockade-responsive pancreatic cancer
Source: Immunother Adv. 2026 Jul 15;6(1):ltag013. doi: 10.1093/immadv/ltag013 (PMC13398997; doi:10.1093/immadv/ltag013)

# Supplemental Figure 1

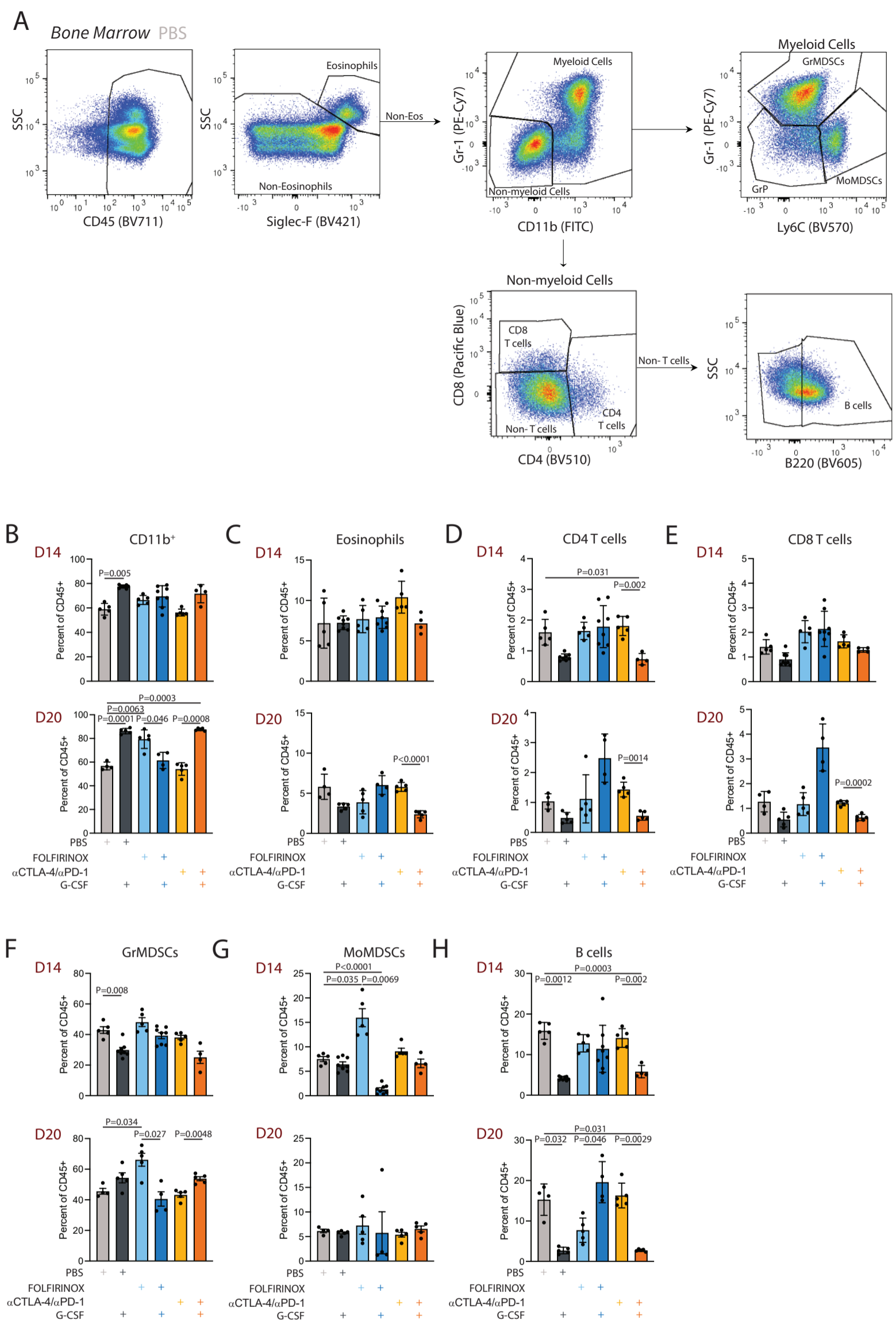

Supplemental Figure 2

A

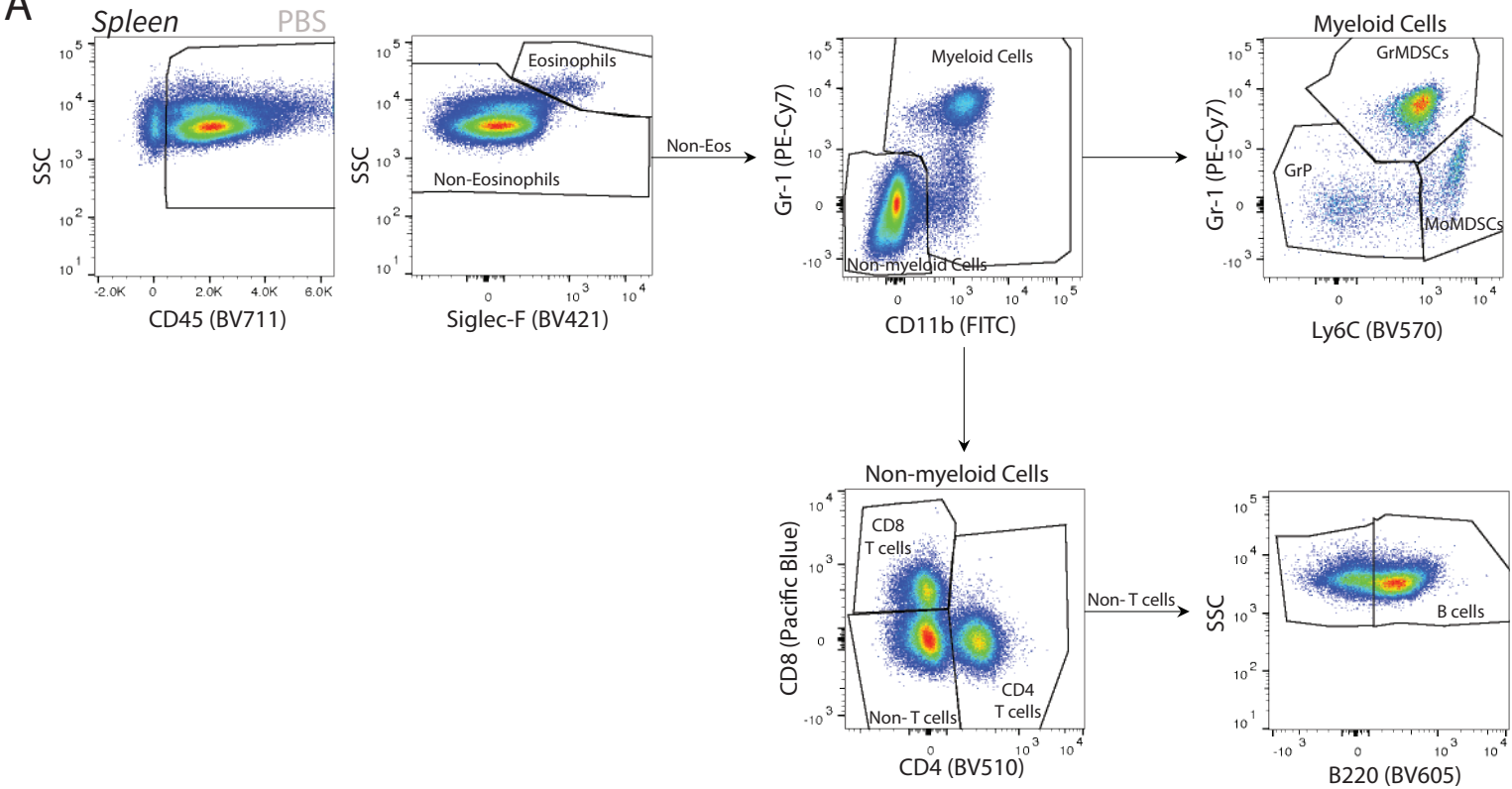

B

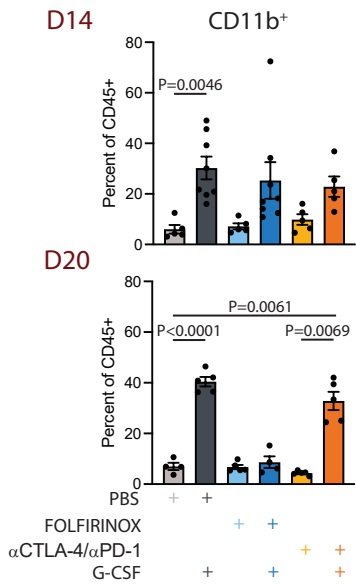

C

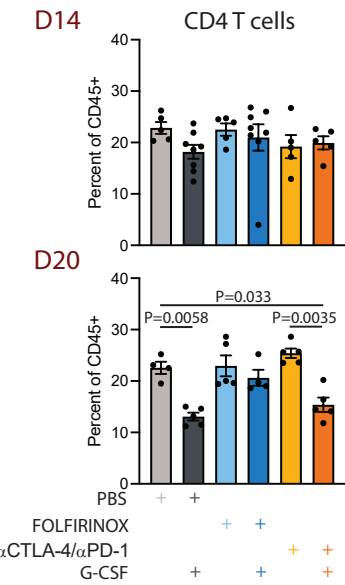

D

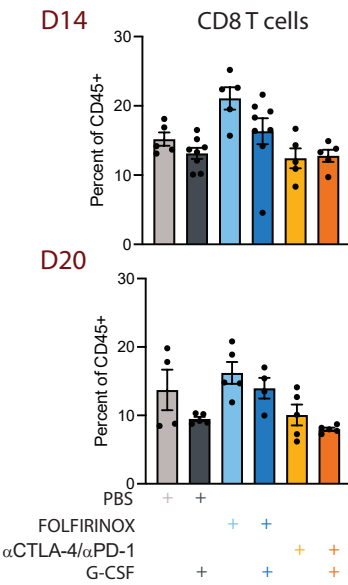

E

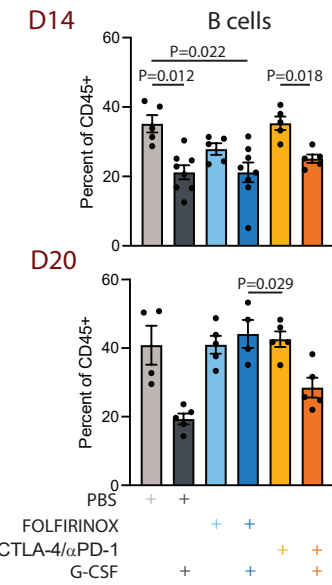

Supplemental Figure 3

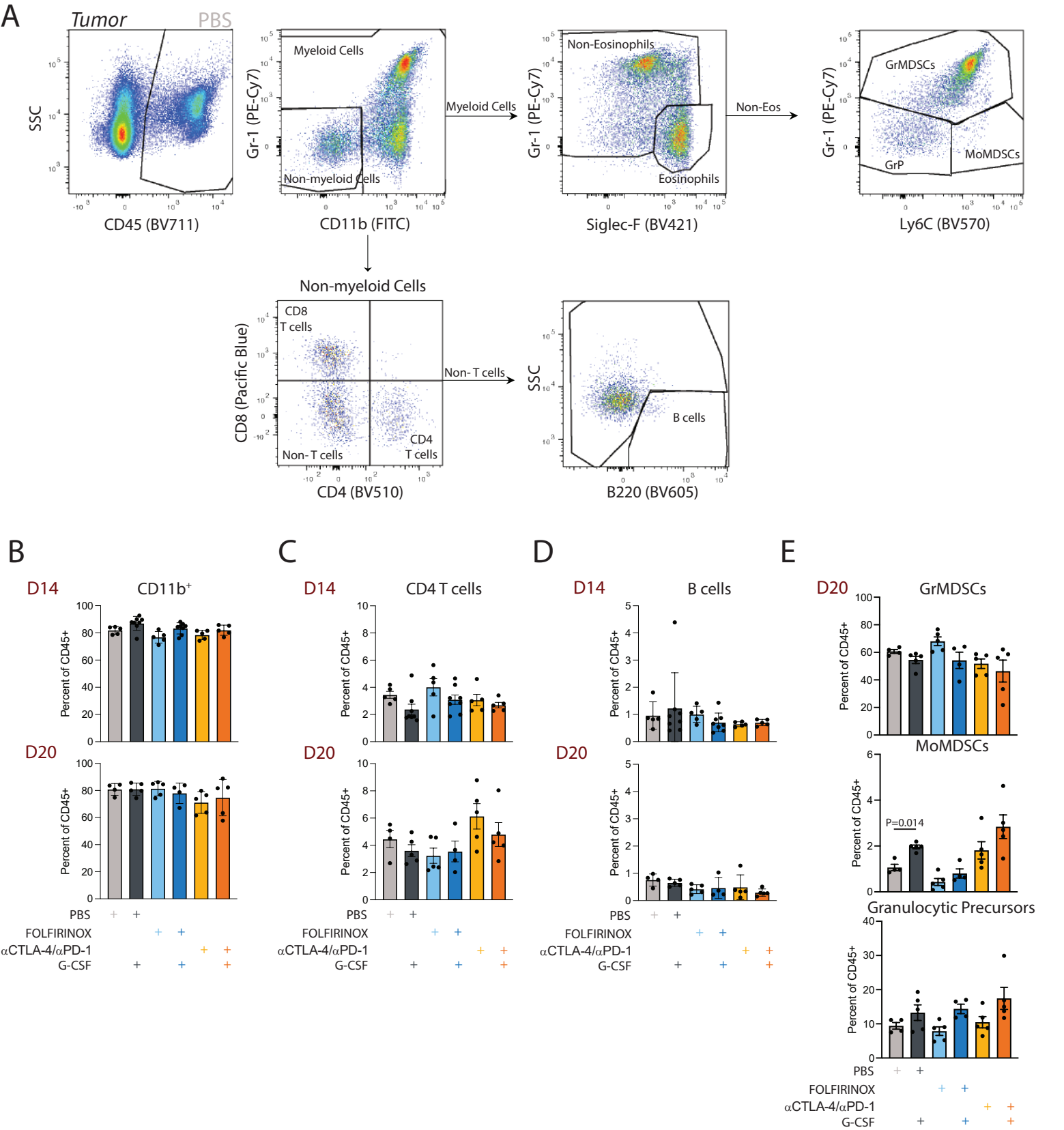

Supplement: ltag013_Supplementary_Data [file ltag013_supplementary_data.zip › Supplemental data combined FINAL.pdf]
